# Hydrophobic GeO_2_ Aerogels by an Epoxide-Induced Process

**DOI:** 10.3390/gels11040225

**Published:** 2025-03-22

**Authors:** Olga M. Gajtko, Svetlana V. Golodukhina, Sergey Yu. Kottsov, Elena N. Subcheva, Vladimir V. Volkov, Gennady P. Kopitsa, Alexandra G. Son, Varvara O. Veselova

**Affiliations:** 1 N.S. Kurnakov Institute of General and Inorganic Chemistry RAS, Leninskii Prosp., 31, Moscow 119991, Russia; brightorangedandelion@gmail.com (S.V.G.); sergey12-17@yandex.ru (S.Y.K.); sonsacha@gmail.com (A.G.S.); ibvarvara@yandex.ru (V.O.V.); 2 Scientific Center for Genetics and Life Sciences, Sirius University of Science and Technology, Olympic Ave 1, Sochi 354340, Russia; subcheva.en@talantiuspeh.ru; 3 Kurchatov Complex of Crystallography and Photonics of NRC «Kurchatov Institute», Leninskii Prosp., 31, Moscow 119333, Russia; volkicras@mail.ru; 4 Konstantinov Petersburg Nuclear Physics Institute of NRC «Kurchatov Institute», Orlova Roshcha 1, Gatchina 188300, Russia; kopitsa_gp@pnpi.nrcki.ru

**Keywords:** epoxide-assisted sol–gel process, gel, aerogel, contact angle, hydrophobicity, luminescence, surface area

## Abstract

This article reports a new straightforward method for producing hydrophobic GeO_2_ aerogels in a one-pot synthesis. For the first time, the epoxide-induced sol–gel process was combined with the co-precursor method to create hydrophobic aerogels. The application of a complex of analytical methods like SEM, TEM, low-temperature nitrogen adsorption–desorption, SAXS and contact angle measurements enabled us to determine that varying the GeCl_4_:(C_2_H_5_)_2_GeCl_2_ ratio allows for targeted adjustments in the morphology, porous structure, and surface properties of aerogels. As the proportion of (C_2_H_5_)_2_GeCl_2_ grows, the surface area increases from 45 to 123 m^2^∙g^−1^ and the contact angle changes from 22.1 to 140.1°. Luminescent properties of the hydrophobic GeO_2_ aerogels are reported for the first time, and it is established that the ratio of green and blue bands in the luminescence spectra when excited under 390 and 235 nm varies depending on the GeCl_4_:(C_2_H_5_)_2_GeCl_2_ ratio used for the aerogel preparation.

## 1. Introduction

Germania aerogels can be promising candidates for lithium-ion battery (LIB) anodes [1,2], catalysts [3,4], optical materials [5,6], etc. However, when compared to other group IV elements, colloidal routes to GeO_2_ gels are much less developed. So far, only gels and aerogels obtained from alkoxide precursors [7,8,9] or by a “liquid glass” route [10] are described in the literature, and those reports are very scarce.

Recently, our research team has developed a methodology for an epoxide-induced process for production of GeO_2_ aerogels with GeCl_4_ as the precursor [11]. The epoxide-induced process is much faster compared to the “liquid glass” procedure and utilizes cheaper and more stable starting materials than the alkoxide route [12]. Unlike other cases reported in the literature, in which predominantly ethanol is used as a solvent, aprotic ethyl acetate was the suitable solvent in this system. Use of this unconventional solvent helped to control the hydrolysis rate, and monolithic transparent gels were successfully formed. The resulting aerogels had a specific surface area of 170 m^2^∙g^−1^, but were hydrophilic. A hydrophilic surface is not desirable for an aerogel, as it makes it susceptible to air humidity and reduces its lifespan. Tuning the wettability of aerogels is a separate synthetic task and several strategies are applicable [13]. Hydrophobic aerogel materials have been prepared using a wide variety of techniques, such as post-synthesis surface modification of the matrix or in situ incorporation of precursors with non-polar substituents into the sol–gel matrix.

The former approach—surface modification or derivatization—is the post-synthetic treatment of the lyogels with alkylchlorosilanes, such as trimethylchlorosilane (TMCS). These compounds interact with the surface of pre-existing nanoparticles and cover them in –CH_3_ or other alkyl groups. This technique was used to prepare the classical silica aerogels from different precursors [14,15,16]; binary ZrO_2_–SiO_2_ aerogels [17]; alumina aerogels [18,19]; magnesium oxide aerogels [20]; and others. As applied to germanium dioxide, our research team has previously reported successful production of hydrophobic GeO_2_ aerogels (contact angle 141° and S_BET_ 90 m^2^∙g^−1^) via modification of GeO_2_ lyogels with methyltrimethoxysilane (MTMS) and subsequent ambient pressure drying [21]. The major drawback of this technique is introduction of foreign silicon atoms into the system, which is not always permissible and for some applications not at all suitable. Substituted alkoxides of other elements are usually less available precursors, and are often unstable. E.g., methyltrimethoxygermane, which could have been used to replace MTMS, is not sold as a pre-made precursor. Besides, the surface modification is a lengthy multi-step process which might take several days.

Compounds which include the hydrophobic non-hydrolysable alkyl groups can be used as precursors in the sol–gel process individually [22], or as a reaction mixture component. Adding some fraction of it, which is sometimes called the “co-precursor method” [23], is more reasonable price-wise, as such compounds are usually more costly than the conventional aerogel precursors. Besides, varying the fraction of the hydrophobic component allows precise control of the wetting angle [24].

However, this approach is well-described only for silica aerogels, and primarily for the alkoxide hydrolysis synthesis. In the present work, we explore the applicability of the co-precursor method for the epoxide-induced synthesis of GeO_2_ aerogels with GeCl_4_ as the primary component and (C_2_H_5_)_2_GeCl_2_ as the hydrophobic additive. The effect of GeCl_4_:(C_2_H_5_)_2_GeCl_2_ ratio on the microstructure and wettability is described. Small-angle X-ray scattering analysis is employed to provide further insight into the mesostructure of the samples.

## 2. Results and Discussion

Diethylgermanium dichloride (C_2_H_5_)_2_GeCl_2_ is a stable compound that does not undergo hydrolysis in various solvents (ethyl acetate, butyl acetate, ethanol and isopropanol). The tests showed that (C_2_H_5_)_2_GeCl_2_ is stable in water and concentrated acids. The hydrolysis reaction occurs only in basic conditions when ammonia solution is added but instead of monolithic gels, GeO_2_ sediments form.

**Figure 1:**
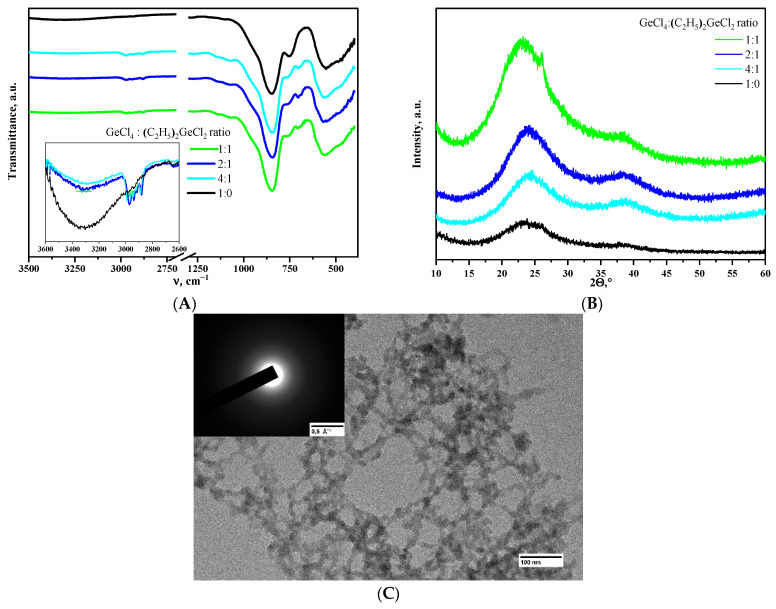
IR spectra (**A**) and XRD patterns (**B**) of aerogels, obtained using different molar ratios of the precursors. Microphotograph and SAED pattern (inlet) of the aerogel prepared using the ratio of GeCl_4_:(C_2_H_5_)_2_GeCl_2_ = 1:1 (**C**).

Incorporating (C_2_H_5_)_2_GeCl_2_ up to the ratio of GeCl_4_:(C_2_H_5_)_2_GeCl_2_ = 1:1 into the epoxide-induced process developed in [11] did not affect the gelation rate, and transparent monolithic gels were obtained successfully. The incorporation of (C_2_H_5_)_2_GeCl_2_ into the 3D network is directly confirmed by the results of IR spectroscopy of aerogels after supercritical drying. The spectrum of aerogel obtained using only GeCl_4_ precursor (Figure 1A) shows intense bands with maxima at 845 and 560 cm^−1^, characteristic of α-GeO_2_ aerogels [8], and a weak wide band of OH-groups in the 3000–3500 cm^−1^ region. IR spectra of aerogel samples prepared with the addition of (C_2_H_5_)_2_GeCl_2_ showed the appearance of small absorption bands in the region of C-H stretching (in the range of 2860–3000 cm^−1^) and deformation vibrations of –CH_2_-CH_3_ groups (1459 and 1380 cm^−1^) [25]. Simultaneously with the appearance of the bands of –CH_2_-CH_3_ groups, there is a relative decrease in the intensity of –OH group vibrations compared to the reference sample prepared without addition of (C_2_H_5_)_2_GeCl_2_.

X-ray phase analysis showed that all the aerogels obtained are amorphous (Figure 1B). The aerogel obtained using the ratio of GeCl_4_:(C_2_H_5_)_2_GeCl_2_ = 1:1 is a minor exception. It is mostly amorphous as well, but a small amount of crystalline GeO_2_ is present according to the XRD data. However, SAED analysis was not able to find the crystalline particles in this particular aerogel (Figure 1C), so this evidence is weak. All the other aerogels studied in the present work were also fully amorphous according to SAED.

**Figure 2:**
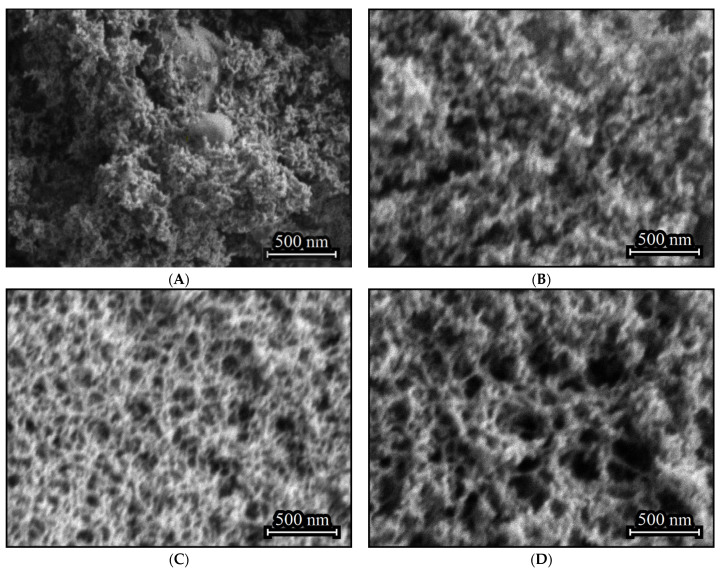
SEM images of aerogel samples prepared with different molar ratios of precursors GeCl_4_:(C_2_H_5_)_2_GeCl_2_: 1:0 (**A**), 4:1 (**B**), 2:1 (**C**) and 1:1 (**D**).

The aerogel obtained in the absence of (C_2_H_5_)_2_GeCl_2_ consists primarily of nanoparticles, with inclusions of larger agglomerates with size up to several hundred nanometers (Figure 2A). Such inclusions do not form if (C_2_H_5_)_2_GeCl_2_ is present in the reaction (Figure 2B–D). Addition of (C_2_H_5_)_2_GeCl_2_ does not significantly affect the particle size, but an increase in its proportion leads to the formation a more developed 3D-structure with larger pores and channels.

**Figure 3:**
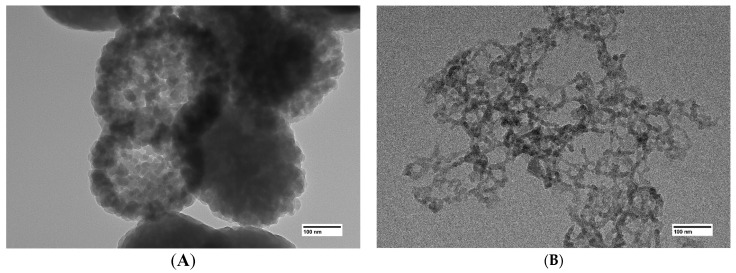
TEM images of aerogel samples prepared with different molar ratios of precursors GeCl_4_:(C_2_H_5_)_2_GeCl_2_: 1:0 (**A**), 1:1 (**B**).

Use of transmission electron microscopy proves that the larger inclusions in (Figure 2A) are in fact dense agglomerations of nanoparticles (Figure 3A). It is likely that particles in the aerogels obtained using the GeCl_4_-(C_2_H_5_)_2_GeCl_2_ mixture are less prone to agglomeration due to the presence of –CH_2_-CH_3_ on the surface. As mentioned earlier, the hydrolysis rate of germanium chloride much exceeds that of (C_2_H_5_)_2_GeCl_2_. Thus, the core of the nanoparticles is formed initially from GeCl_4_ and is later covered by products of (C_2_H_5_)_2_GeCl_2_ hydrolysis.

**Figure 4:**
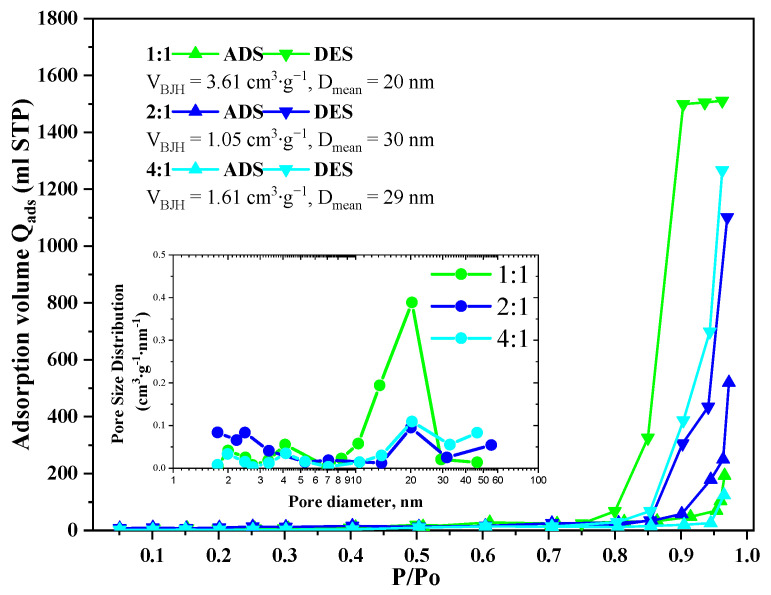
Isotherms of low-temperature adsorption–desorption of nitrogen in GeO_2_ aerogel samples prepared with different molar ratios of precursors GeCl_4_:(C_2_H_5_)_2_GeCl_2_.

Nitrogen adsorption-desorption isotherms of the GeO_2_ aerogels belong to Type 3 according to IUPAC with H2(b) type hysteresis in the case of the 1:1 ratio aerogel and with H3 type in the case of the 2:1 and 4:1 ratio aerogels (Figure 4). The pore size distributions calculated using the Barrett–Joyner–Halenda model indicate the presence of 10–30 nm mesopores in all samples. In the aerogels prepared with 2:1 and 4:1 precursor ratio, some macropores with size above 50 nm are present, though the bigger fraction of pores remains in the 15–30 nm range. The cumulative pore volume for all samples lies within 1.05–3.61 cm^3^/g. The largest cumulative pore volume is characterized for the aerogel prepared with 1:1 precursor ratio, which agrees well with SEM data (Figure 2D).

**Figure 5:**
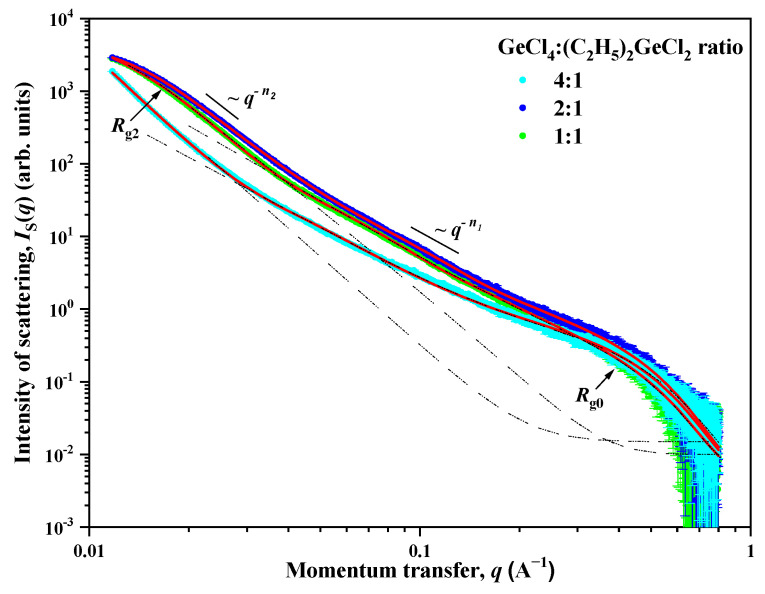
Dependences of the intensity *I*_S_(*q*) of small-angle X-ray scattering on the momentum transfer *q* for aerogel samples prepared with different molar ratios of GeCl_4_:(C_2_H_5_)_2_GeCl_2_ precursors. Solid lines are the result of the description of experimental data using Formula (1).

To provide further insight into the mesostructure of the samples, small-angle X-ray scattering analysis was employed. The experimental dependences of the intensity *I*_S_(*q*) of small angle X-ray scattering for aerogel samples prepared with the use of (C_2_H_5_)_2_GeCl_2_ are presented in double logarithmic scale in Figure 5. A common feature for all the studied samples is the presence of two ranges for the momentum transfer *q* with a crossover point *q*_c_ (point of crossover from one scattering regime to another) between them on the corresponding scattering curves. The behavior of the intensity *I*_S_(*q*) of SAXS follows the power laws *q*^−Δ^ with different values Δ = *n*_1_ and *n*_2_. In addition, a so-called “shoulder” is present on all the scattering curves in the region of *q* > 0.3 Å, which indicates the presence of small spherical inhomogeneities (particles) with a characteristic size of *R*_0_. The behavior of the scattering intensity *I*_S_(*q*) in this case is described by the Guinier approximation with $R_{0}=\sqrt{5/3}R_{g0}$ [26].

The values of the exponent *n*_1_, found from the slope of the rectilinear sections of the experimental *I*_S_(*q*) curves, range from 2.50 to 2.60. This corresponds to scattering on inhomogeneities (clusters) structured according to the type of mass fractal with dimensions of 2.50 ≤ *D*_M1_ = *n*_1_ ≤ 2.60 [27], and with the lower limit of self-similarity, which is determined by the size *R*_0_ of the small spherical inhomogeneities (particles) which were described earlier. The upper bound of self-similarity *R*_c1_ for mass fractal inhomogeneities (clusters) cannot be determined from the presented data due to the superposition (at *q* < *q*_c_) of the contribution of scattering on large-scale inhomogeneities described by the power dependence *q*^−*n*2^. However, it can be argued that the values of the upper bound of self-similarity *R*_c1_ exceed the values obtained from the ratio *R*_c_ = π/*q*_c_. The values of the power coefficient *n*_2_ for all aerogels are close to 4 (Porod’s law), which corresponds to scattering on objects with an almost smooth surface *D*_S2_ = 6 − *n*_2_ ≈ 2 [27].

In samples prepared with a molar ratio of GeCl_4_:(C_2_H_5_)_2_GeCl_2_ = 2:1 and 1:1, a deviation from the *q*^−*n*2^ power dependence is observed in the region of small *q*. This deviation indicates the presence of large-scale inhomogeneities (aggregates) with a characteristic size of the *R*_c2_. The absence of this deviation for a sample with a molar ratio of 4:1, in turn, means that the characteristic size of *R*_c2_ exceeds the maximum size of inhomogeneities *R*_max_ ≈ 3.5/*q*_min_ [28], the scattering from which can be recorded experimentally at a given resolution of the device. In the case of the SAXS device used for the study, it is *q*_min_ = 0.01 Å^−1^ and *R*_c2_ > *R*_max_ ≈ 350 Å.

Thus, the scattering pattern observed for aerogel samples is typical for scattering in systems with a disordered structure and indicates that in these samples there are several types of scattering inhomogeneities that differ greatly in type and their characteristic scale *R*_c_. To account for that, the following expression was used to analyze the scattering by aerogel samples. This expression takes into account the presence of three types of scattering inhomogeneities in the system:

(1) $$I_{S}(q)=\left\{\begin{array}{l} \frac{B_{2}}{{\left(q^{2}+\kappa^{2}\right)}^{\frac{n_{2}}{2}}},\;\;\;\;\;\;\;q<q_{c}\\ \frac{B_{1}}{q^{n_{1}}}+G_{0}\cdot \exp(\frac{q^{2}R_{g0}^{2}}{3})+I_{inc},\;q>q_{c} \end{array}\right.,$$

where *G*_0_ is the Guinier pre-factor for small spherical inhomogeneities, *B*_1_ and *B*_2_ are exponential pre-factors which depend on the local structure of the scattering inhomogeneities [29], *κ* = 1/*R*_c2_ is the inverse correlation radius, and *n*_1_ and *n*_2_ are exponents. The *I_inc_* parameter is a constant defined by incoherent scattering from inhomogeneities of the order of the wavelength of the radiation used and is independent of *q*.

To obtain the final results, expression (1) was convoluted with the function of the device resolution, and then processed using the least squares method. The processing results are shown in Figure 5, as well as in Table 1.

According to the data presented in Table 1, it can be seen that all the studied aerogels are porous systems with a disordered structure and consist of small spherical inhomogeneities (particles) with a size of *d*_0_ = 2*R*_0_ ≈ 1.2 nm, from which mass-fractal inhomogeneities (clusters) are formed. The fractal dimension of these clusters decreases from *D*_M1_ = 2.60 (4:1 reactant ratio) to 2.50 (1:1 reactant ratio). This indicates a “thinning out” of the structure with an increase in the content of (C_2_H_5_)_2_GeCl_2_ in the reaction mixture, which is in good agreement with SEM and TEM data (Figure 2 and Figure 3). These aerogels also contain large-scale inhomogeneities (aggregates) with an almost smooth phase interface (solid phase—pore), the characteristic size of which *d*_2_ = 2*R*_2_ decreases significantly with an increase in the amount of (C_2_H_5_)_2_GeCl_2_ in the reaction mixture: >70 nm (4:1) to ≈18 (2:1) and ≈22 (1:1) nm, respectively. The values obtained using the SAXS method slightly differ from the values obtained from the isotherms of low-temperature adsorption–desorption of nitrogen, but both methods show decrease in the pore size and increase in the surface with increasing share of (C_2_H_5_)_2_GeCl_2_ in the precursor ratio.

**Figure 6:**
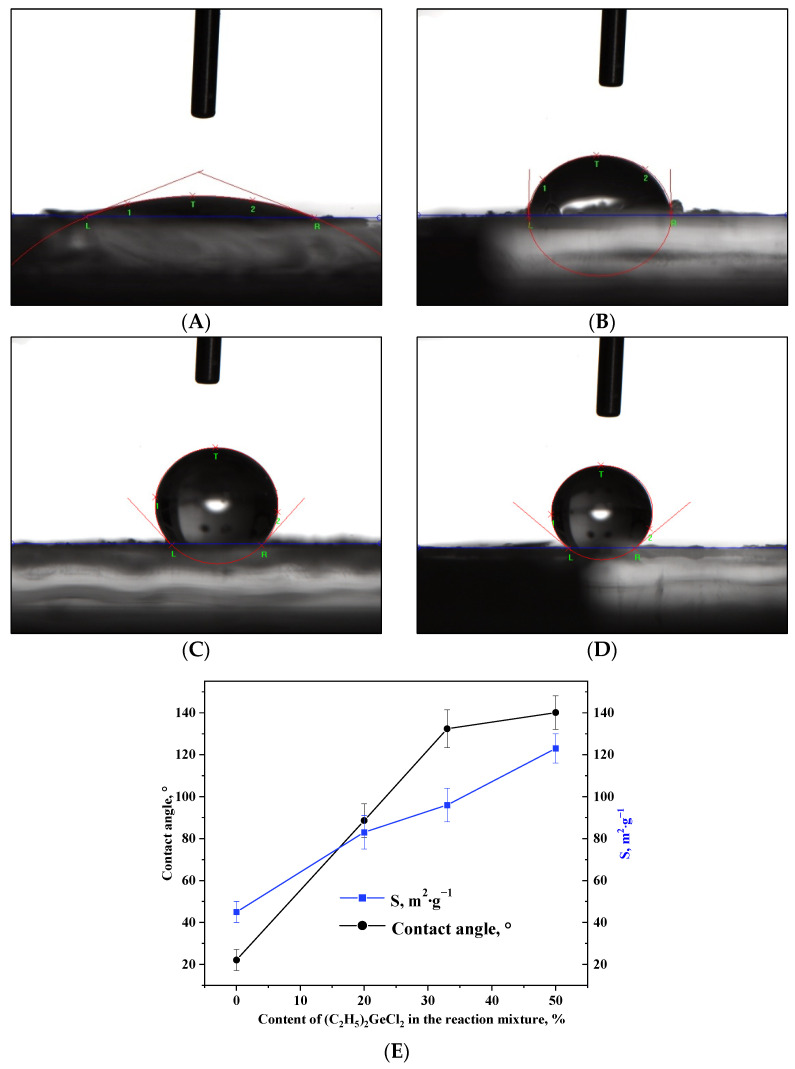
Photos of a water droplet on the surface of aerogel samples prepared with different molar ratios of precursors GeCl_4_:(C_2_H_5_)_2_GeCl_2_: 1:0 (**A**), 4:1 (**B**), 2:1 (**C**) and 1:1 (**D**). Dependence of the contact angle and specific surface area on the content of (C_2_H_5_)_2_GeCl_2_ in the reaction mixture (**E**).

The change in porous structure upon the addition of (C_2_H_5_)_2_GeCl_2_ into the reaction mixture is accompanied by a nearly three-fold increase in the surface area (Table 2, Figure 6E). This enlargement of specific surface area can be attributed to the increased quantity of non-polar alkyl groups (–CH_2_-CH_3_) on the surface of the aerogels, which reduces shrinkage during drying and therefore increases specific surface area. The –CH_2_-CH_3_ groups present on the surface of the nanoparticles play a key role in the formation of the more developed 3D network. At the same time, such groups have been shown to affect the wetting of oxide aerogels prepared via alkoxide hydrolysis with addition of hydrophobic precursors. Measurement of the contact angles of the aerogels prepared in the course of the present work proved that this technique is applicable in the case of the epoxide-assisted process as well (Figure 6A–E). The aerogel synthesized without the addition of (C_2_H_5_)_2_GeCl_2_ had a hydrophilic surface (Figure 6A); as the proportion of (C_2_H_5_)_2_GeCl_2_ increased, the surface gradually hydrophobized, and already at the ratio of GeCl_4_:(C_2_H_5_)_2_GeCl_2_ = 2:1, the aerogel had a hydrophobic surface.

**Figure 7:**
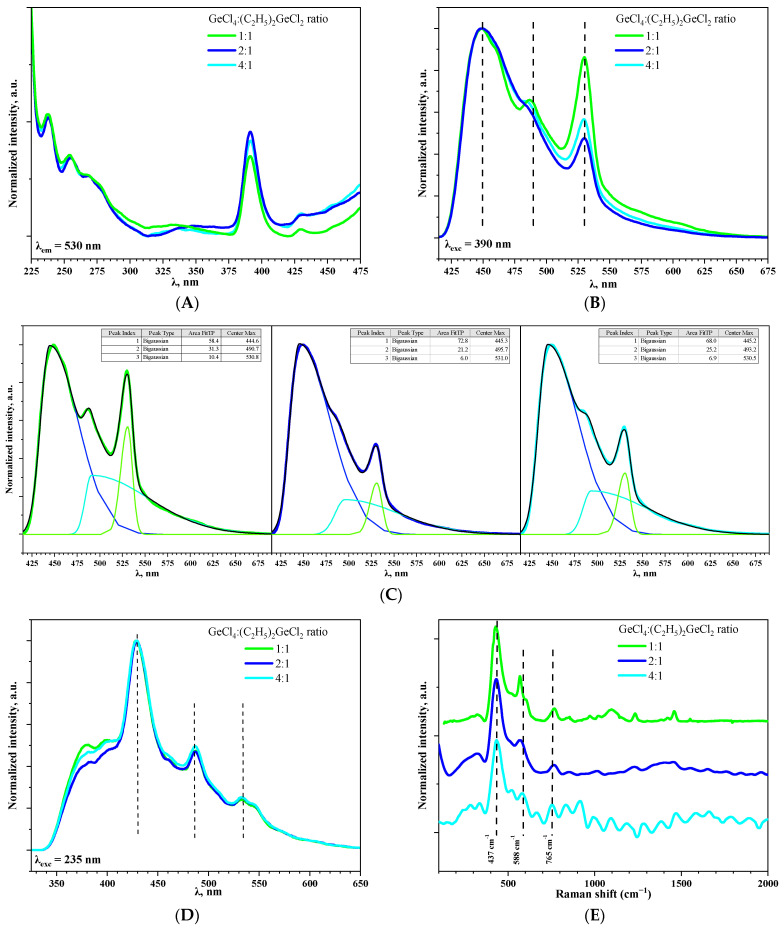
The photoluminescence excitation spectra of aerogel samples prepared with different molar ratios of precursors GeCl_4_:(C_2_H_5_)_2_GeCl_2_ recorded at 530 nm (**A**). The photoluminescence spectra of aerogel samples prepared with different molar ratios of precursors GeCl_4_:(C_2_H_5_)_2_GeCl_2_ at λ_exc_= 390 (**B**) and 235 (**D**) nm. Bi-Gaussian decomposition of spectrum, presented in Figure 7B (**C**). Raman spectra of aerogel samples prepared with different molar ratios of precursors GeCl_4_:(C_2_H_5_)_2_GeCl_2_ (**E**).

Excitation spectra for aerogel samples prepared with different molar ratios of precursors GeCl_4_:(C_2_H_5_)_2_GeCl_2_ were registered at 530 nm (Figure 7A). Spectra for all samples are similar and the most prominent band was observed at 390 nm. This is in good agreement with luminescence spectra of GeO_2_ reported in [10].

On the photoluminescence spectra of aerogels recorded under excitation at 390 nm, three bands at 445, 490 (as shoulder) and 530 nm could be observed (Figure 7B). It is considered that the emission of blue and green light is a result of radiative recombination processes associated with defects, such as oxygen and oxygen–germanium vacancies, in GeO_2_ crystals [30]. Moreover, the band at 530 nm is more typical for commercial bulk GeO_2_ powder [31], while the band at 448 nm is more common for highly oxygen-deficient GeO_2_ nanoparticles [32]. The ratio of band intensities varied depending on GeCl_4_:(C_2_H_5_)_2_GeCl_2_ proportion; the most significant contribution of green light emission (530 nm) is observed when the ratio is 1:1 (Figure 7C). One of the possible reasons could be the presence of small amount of crystalline GeO_2_ in this aerogel (Figure 1B). It might also be suggested that use of (C_2_H_5_)_2_GeCl_2_ somehow affects the oxygen stoichiometry of the prepared aerogels, which causes differences in the luminescence intensity. However, this dependency is non-linear, since for the aerogel prepared with the “medium” ratio of GeCl_4_:(C_2_H_5_)_2_GeCl_2_ = 2:1, the band at 530 nm is the least intense. Interestingly, with an increase in the excitation energy (λ_exc_ = 235 nm), the ratio of the intensity of the bands at 530, 490, and 445 nm became nearly equal in all three samples (Figure 7D). Simultaneously, two additional weak emission bands emerged at 397 and 380 nm, for which the dependence observed during excitation under 390 nm repeats. M.S. Rathore et al. [33] reported violet photoluminescence for GeO_2_ and its origin was attributed to the neutral oxygen vacancy centers.

The presence of non-bridging oxygen could be indirectly confirmed by Raman spectroscopy and the presence of oxygen deficiencies could be estimated this way (Figure 7E) [34]. However, it was impossible to obtain Raman spectra with high resolution due to strong luminescence of the aerogels. An intense band with a maximum at 437 cm^−1^ corresponds to typical symmetric valence vibrations of Ge-O-Ge in four-membered rings consisting of [GeO_4_] tetrahedra [10] and the band at 588 cm^−1^ corresponds to deformation vibrations of the Ge-O-Ge tetrahedron [GeO_4_] [35]. Increase in (C_2_H_5_)_2_GeCl_2_ proportion led to the shift of this band to the short-wave region. The vibrational modes in the range above 700–1500 cm^−1^ are assigned to asymmetric stretching vibration of Ge-BO-Ge (BO—bridging oxygen) and symmetric and asymmetric Ge-NBO (NBO—non-bridging oxygen) vibrations. The presence of these bands evidences high concentration of defects in the prepared aerogels [34].

**Figure 8:**
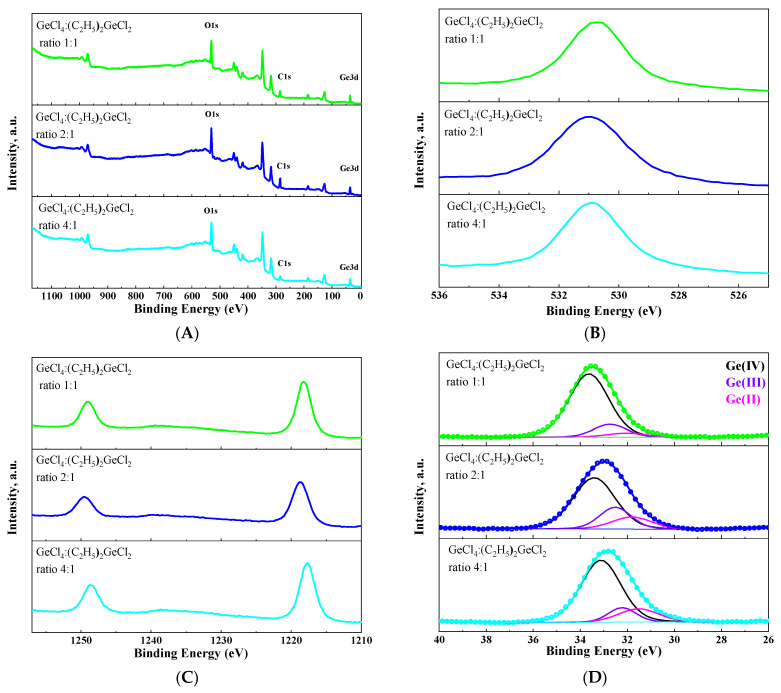
XPS survey spectra (**A**) and O1s (**B**), Ge2p (**C**) and Ge3d (**D**) XPS spectra of aerogel samples prepared with different molar ratios of precursors GeCl_4_:(C_2_H_5_)_2_GeCl_2_.

The chemical valence state of germanium on the surface of the obtained aerogel has been analyzed by XPS (Figure 8). As expected, in addition to germanium and oxygen transitions, a carbon transition could be observed in XPS survey spectra for all samples (Figure 8A). This provides additional evidence that surface modification has been successfully carried out. The Ge (3d) peak was split into three components corresponding to Ge^2+^, Ge^3+^ and Ge^4+^ states [36,37,38] for all aerogels (Figure 8D). This confirms the assumption that aerogels produced under these conditions are oxygen-deficient. Similarly to the luminescent properties, the proportion of each germanium state on the surface of the aerogel depended non-linearly on the ratio of GeCl_4_:(C_2_H_5_)_2_GeCl_2_ in the initial mixture of precursors. Moreover, an increase in the intensity of green luminescence at 530 nm directly correlates with an increase in the proportion of Ge (IV) on the surface of the aerogel.

## 3. Conclusions

A new simple low-effort method for the production of GeO_2_ aerogels is developed to replace the alkoxide process and reduce the material cost. For the first time, successful combination of the epoxide-assisted process and the co-precursor approach is reported. The possibility of modifying the properties of an aerogel is demonstrated using a set of complementary analytical methods. Changes in the particle size and porous structure depending on the proportion of (C_2_H_5_)_2_GeCl_2_ in the precursor mixture are described. At the maximum used ratio of 1:1 GeCl_4_:(C_2_H_5_)_2_GeCl_2_ a hydrophobic aerogel with wetting angle of 140° is produced. Hydrophobicity is known to prolong service time of the aerogel and the ability to produce hydrophobic aerogels in a single-step process is a significant advantage of the developed method.

Dependence of the luminescent properties of the GeO_2_ aerogel on the porous structure and particle size is reported for the first time. The introduction of diethylgermanium dichloride into the system affects the defectiveness and oxygen deficiency of the aerogel surface, which is manifested in a change in the ratio of the green and blue bands in the photoluminescence spectra.

The data on luminescent properties of GeO_2_ aerogels can be used to develop new optical materials. The new synthetic route can also be employed for production of LIB anodes and catalyst supports with custom porous structure depending on the exact task at hand.

## 4. Materials and Methods

### 4.1. Synthesis of the Gels

The following reagents were used: GeCl_4_ (Sigma-Aldrich, St. Louis, MO, USA, 99.99%), (C_2_H_5_)_2_GeCl_2_ (Sigma-Aldrich, St. Louis, MO, USA, 97%), ethyl acetate (Himmed, Moscow, Russia, high grade), propylene oxide (Sigma-Aldrich, St. Louis, MO, USA, 99.5%), hydrochloric acid (Sigma Tech, Khimki, Russia, high grade).

To obtain a gel with GeCl_4_:(C_2_H_5_)_2_GeCl_2_ ratio of 1:1, first, 0.1 mL (0.87 mmol) of GeCl_4_ and 0.128 mL (0.87 mmol) of (C_2_H_5_)_2_GeCl_2_ were dissolved in 5.1 mL (52.2 mmol) of ethyl acetate, 0.609 mL (8.7 mmol) of propylene oxide was added to the mixture, and the resulting solution was cooled at −18 °C for 20 min. The obtained solution was placed in a polypropylene container and 0.081 mL of 36.5 mass.% hydrochloric acid (0.92 mmol) was added under ultrasonic treatment. The molar ratio of reagents in the system GeCl_4_:(C_2_H_5_)_2_GeCl_2_:ButAc:HCl:Propylene oxide was 1:1:60:1.06:10. The gel formed within two minutes.

A series of samples with different ratios of GeCl_4_ to (C_2_H_5_)_2_GeCl_2_ was obtained; the ratios were equal to 4:1, 2:1 and 1:1, and the quantity of GeCl_4_ was always kept at 0.87 mmol when these ratios were calculated.

### 4.2. Supercritical Drying in CO_2_

To perform supercritical drying, the lyogel was placed in a 50 mL steel reactor and washed with liquid CO_2_ for 2 h at 20 °C and 15 MPa. After this initial step, the temperature was increased to 50 °C and the sample was washed with supercritical CO_2_ for 2–2.5 h. A high-pressure CO_2_ pump Supercritical 24 (SSI, Chicago, IL, USA) and a back pressure regulator BPR (Goregulator, Waters, Milford, MA, USA) were employed to maintain the target conditions.

### 4.3. X-Ray

The phase composition of the produced aerogels was studied using a Bruker (Billerica, MA, USA) D8 Advance X-ray diffractometer (CuKa radiation, Ni filter and Lynxeye detector) using a step length of 0.02° and time per step of 0.3 s. The phase composition was determined based on comparison with the powder diffraction standard files in the PDF2 database.

### 4.4. IR Spectroscopy

FTIR spectra analysis was performed using an InfraLUM FT-08 spectrometer (Lumex, St.Petersburg. Russia) in the attenuated total reflectance (ATR) mode with a Specac diamond attachment. The spectra were acquired in the range of 400–4000 cm^−1^ with a spectral resolution of 2 cm^−1^ and averaging 40 spectra.

### 4.5. Electron Microscopy

A Tescan Amber GMH high-resolution electron microscope (Tescan Orsay Holding, a.s., Brno–Kohoutovice, Czech Republic) and a JEOL JEM 2100Plus HR transmission electron microscope (JEOL, Tokyo, Japan) were used to study the microstructure of the aerogels. To obtain the TEM images, the samples were dispersed in ethanol and placed onto copper formvar/carbon TEM grids (Ted Pella Inc., Redding, CA, USA). The TEM microphotographs and the corresponding selected area electron diffraction (SAED) patterns were acquired in brightfield mode at an acceleration voltage of 200 kV.

### 4.6. Wetting Angle

The contact angles were measured on an FT A 200 device (First Ten Angstroms, Inc., Portsmouth, VA, USA). The resulting photos were processed using FT200 software.

### 4.7. Low-Temperature Nitrogen Adsorption–Desorption

The specific surface area and pore size distribution were analyzed using an ATX-06 analyzer (Katakon, Novosibirsk, Russia) and calculated as described in [21].

### 4.8. Luminescent Properties

A PerkinElmer LS-55 single-beam luminescence spectrometer (PerkinElmer, Inc., Waltham, MA, USA) was used to acquire luminescence spectra of the aerogels at λ_em_ = 530 nm and at λ_exc_ = 390, 360, 280, 255 and 235 nm; the optical slit sizes were 10 nm, and resolution of the setup was 0.5 nm. The spectra were acquired at room temperature.

### 4.9. Raman Spectroscopy

A setup based on a Confotech NR-500 spectrometer (Sol Instruments, Augsburg, Germany), described in detail in [10], was used to acquire the Raman spectra.

### 4.10. Small Angle X-Ray Scattering (SAXS)

The SAXS experiment was carried out using a setup based on the AMUR-K diffractometer and described in detail in [39] at Kurchatov Complex of Crystallography and Photonics of NRC «Kurchatov Institute» (Moscow, Russia). The aerogels were put in a vacuum chamber at 700 mm from the detector. The beam cross-section was 8 × 0.2 mm, the range of scattering angles *θ* corresponded to the *q*-range from 1.0 10^−2^ to 1 Å^−1^. The measurement time per sample amounted to 10 min. Experimental data were normalized to the incident beam intensity, after which they were corrected for collimation distortions.

### 4.11. X-Ray Photoelectron Spectroscopy

X-ray photoelectron spectra (XPS) were recorded using the PREVAC EA15 electronic spectrometer (PREVAC, Rogów, Poland) and AlKα radiation source (hν = 1486.74 eV, 150 W). CasaXPS Software Version 2.3.15 was used for spectra deconvolution.

## Figures and Tables

**Table 1:** Parameters of mesostructure for aerogel samples obtained from analysis of SAXS data.

Parameters	Molar Ratio GeCl_4_:(C_2_H_5_)_2_GeCl_2_		
	4:1	2:1	1:1
Third type of structural inhomogeneities			
*R*_c2_ = 1/*κ*, Å	>350	90 ± 10	110 ± 10
*n*_2_	4.02 ± 0.02	4.00 ± 0.02	3.98 ± 0.02
Second type of structural inhomogeneities			
*R*_c1_ = π/*q*_c_ (Å)	>90	>70	>80
*D*_M1_ = *n*_1_	2.60 ± 0.02	2.53 ± 0.02	2.50 ± 0.02
First type of structural inhomogeneities			
*R*_c0_ = √5/3 *R*_g0_ (Å)	6.0 ± 0.5	6.0 ± 0.5	6.0 ± 0.5

**Table 2:** Dependence of the contact angle and specific surface area on the molar ratio of the precursors GeCl_4_:(C_2_H_5_)_2_GeCl_2_.

Molar Ratio GeCl_4_:(C_2_H_5_)_2_GeCl_2_	1:0	4:1	2:1	1:1
**S, m^2^∙g^−1^ (BJH)**	45	83	96	123
**Contact angle, °**	22.1	88.6	132.4	140.1

## Data Availability

The original contributions presented in the study are included in the article; further inquiries can be directed to the corresponding author.
